# Neural Activity Predicts Reaction in Primates Long Before a Behavioral Response

**DOI:** 10.3389/fnbeh.2018.00207

**Published:** 2018-09-11

**Authors:** Mohsen Parto Dezfouli, Mohammad Bagher Khamechian, Stefan Treue, Moein Esghaei, Mohammad Reza Daliri

**Affiliations:** ^1^Neuroscience and Neuroengineering Research Laboratory, Department of Biomedical Engineering, School of Electrical Engineering, Iran University of Science and Technology, Tehran, Iran; ^2^Cognitive Neuroscience Laboratory, German Primate Center-Leibniz Institute for Primate Research, Goettingen, Germany; ^3^Faculty of Biology and Psychology, University of Goettingen, Goettingen, Germany; ^4^Bernstein Center for Computational Neuroscience, Goettingen, Germany; ^5^Leibniz-Science Campus Primate Cognition, Goettingen, Germany; ^6^Cognitive Neurobiology Laboratory, School of Cognitive Sciences, Institute for Research in Fundamental Sciences, Tehran, Iran

**Keywords:** reaction time, prediction, attention, visual area MT, electrophysiology, macaque monkey

## Abstract

How neural activity is linked to behavior is a critical question in neural engineering and cognitive neurosciences. It is crucial to predict behavior as early as possible, to plan a machine response in real-time brain computer interactions. However, previous studies have studied the neural readout of behavior only within a short time before the action is performed. This leaves unclear, if the neural activity long before a decision could predict the upcoming behavior. By recording extracellular neural activities from the visual cortex of behaving rhesus monkeys, we show that: (1) both, local field potentials (LFPs) and the rate of neural spikes long before (>2 s) a monkey responds to a change, foretell its behavioral performance in a spatially selective manner; (2) LFPs, the more accessible component of extracellular activity, are a stronger predictor of behavior; and (3) LFP amplitude is positively correlated while spiking activity is negatively correlated with behavioral reaction time (RT). These results suggest that field potentials could be used to predict behavior way before it is performed, an observation that could potentially be useful for brain computer interface applications, and that they contribute to the sensory neural circuit’s speed in information processing.

## Introduction

How neural activities control the behavior (either choice or performance) is a crucial question in neuroscience. Previous studies have reported a link between behavioral performance and the neural activity in different brain areas, such as the trigeminothalamic system (Maixner et al., 1989; Bushnell et al., 1993), the somatosensory system (Sinclair and Burton, 1991; Romo et al., 1996), superior colliculus (Horwitz and Newsome, 2001) and visual cortex (Newsome et al., 1989; Britten et al., 1996; Shadlen et al., 1996; Horwitz and Newsome, 2001; Cook and Maunsell, 2002; Barberini et al., 2005; Liu and Newsome, 2005, 2006; Cohen and Newsome, 2009; Cohen and Maunsell, 2010; Kajikawa and Schroeder, 2011; van Ede et al., 2012; Smith et al., 2015). Additionally, it has been reported that behavioral choice is closely connected to the neural activity (Johansson and Vallbo, 1979; Newsome et al., 1989; Bushnell et al., 1993; Britten et al., 1996; Romo et al., 1996; Shadlen et al., 1996; Horwitz and Newsome, 2001; Cook and Maunsell, 2002; Barberini et al., 2005; Liu and Newsome, 2005, 2006).

Newsome et al. (1989) showed that the behavioral choice could be derived from the relative discharge activity of some direction-selective MT neurons. Other studies have documented that the trial-by-trial variability of an MT neuron’s response is correlated with the monkey’s choice (Britten et al., 1996; Barberini et al., 2005). While many studies have reported a link between behavioral choice and the activity of single neurons, some others suggest that perceptual coding should be described in terms of the pooled activity of neuronal populations (Britten et al., 1996; Liu and Newsome, 2005; Cohen and Newsome, 2009). For instance, Cohen and Newsome (2009) suggested, that to understand the neural mechanisms underlying a behavioral choice, one needs to consider the dynamics of the neural population activity in time. Besides, Liu and Newsome (2005) showed that the behavioral choices are correlated with the neural activity pooled across populations of neurons.

Many such studies focused on the neural spiking activity in order to predict the behavioral choice of subjects, however how the local field potential (LFP) represents this component of behavior has not been studied well. LFP is believed to consist of local synaptic and volume conducted potentials (Kajikawa and Schroeder, 2011), and is widely used as a measure of neural interaction (Ray and Maunsell, 2011; Ray, 2015). Neurophysiologists have frequently used the LFP to link the neural activity to perception and cognition (Gail et al., 2004; Liu and Newsome, 2006; Womelsdorf et al., 2006). Liu and Newsome (2006) reported that an LFP’s temporal variability is highly correlated with multiunit activity of MT neurons and also a monkey’s perceptual choice, especially for frequencies above 40 Hz. Furthermore, the mechanism through which cognitive functions such as attention modulate perception and behavioral performance is not clear. Visual attention enhances perception for an attended stimulus and also modulates the responses of neurons in the visual cortex (Desimone and Duncan, 1995; Luck et al., 1997; Reynolds and Chelazzi, 2004; van Ede et al., 2012; Esghaei et al., 2015, 2018; Xue et al., 2016). Furthermore, there are reports that attention modulates the link between the neural activity and perceptual capability (Cook and Maunsell, 2002; Womelsdorf et al., 2006; Cohen and Maunsell, 2010; van Ede et al., 2012; Mayo et al., 2015). For instance, an instantaneous measure of attention on a few dozen simultaneously recorded V4 neurons could predict the fluctuation of animal’s behavioral performance (Cohen and Maunsell, 2010; Mayo et al., 2015). Therefore, understanding the correspondence between neural activity and behavioral performance helps to understand how cognitive functions such as attention may influence behavior.

Behavioral reaction time (RT) has been a widely used measure of perceptual performance (Meyer et al., 1988; Grondin, 2010; Medina et al., 2015). Cook and Maunsell (2002) found that the neuronal activity in MT and ventral intra-parietal (VIP) areas are correlated with the monkey’s detection RT on a trial-by-trial basis. Also Bell et al. (2006) suggested that the reduction in saccadic response time observed following high-intensity visual stimuli is due to the reduction of processing time along the visual pathway. Furthermore, Lakatos et al. (2008) showed that a rhythmic presence of stimuli entrains the delta-band neural oscillation in cortex and consequently controls the RT according to the oscillation phase. Womelsdorf et al. (2006) demonstrated that RT is positively correlated with the degree of the gamma-band synchronization of V4 neurons, just before the change onset. While all these studies have focused on the neural activity surrounding the behaviorally relevant visual target (a stimulus or its change), it is not clear whether and how the neural activity long before the visual target predicts the behavioral performance.

Although previous studies confirmed the relationship between the behavioral response and neural activity (either LFP or spiking activity), they all found this link only directly before the behaviorally relevant event. Here, we investigate if RT could be predicted by the neural activity long before the behaviorally relevant event is presented to the subject, and how LFP or spiking activity might differ in their link to RT. Therefore, we train rhesus monkeys to perform a visual detection task and record the neural signals from their visual cortex (area MT), while they perform the task. By separately focusing on trials with different behavioral responses, we examine how early before the target visual event, these neural activities could predict the animal’s RT.

## Materials and Methods

### Animal Welfare

Research with non-human primates represents a small but indispensable component of neuroscience research. The scientists in this study are aware and are committed to the great responsibility they have in ensuring the best possible science with the least possible harm to the animals (Roelfsema and Treue, 2014). All animal procedures of this study have been approved by the responsible regional government office (Niedersaechsisches Landesamt fuer Verbraucherschutz und Lebensmittelsicherheit (LAVES)) under the permit numbers 33.42502/08-07.02 and 33.9.42502-04-064/07. The animals were group-housed with other macaque monkeys in facilities of the German Primate Center in Goettingen, Germany in accordance with all applicable German and European regulations. The facility provides the animals with an enriched environment (including a multitude of toys and wooden structures; Calapai et al., 2017; Berger et al., 2018), natural as well as artificial light, exceeding the size requirements of the European regulations, including access to outdoor space. Surgeries were performed aseptically under gas anesthesia using standard techniques, including appropriate peri-surgical analgesia and monitoring to minimize potential suffering (Pfefferle et al., 2018).

The German Primate Center has several staff veterinarians that regularly monitor and examine the animals and consult on procedures. During the study the animals had unrestricted access to food and fluid, except on the days where data were collected or the animal was trained on the behavioral paradigm. On these days the animals were allowed unlimited access to fluid through their performance in the behavioral paradigm. Here the animals received fluid rewards for every correctly performed trial. Throughout the study the animals’ psychological and veterinary welfare was monitored by the veterinarians, the animal facility staff and the lab’s scientists, all specialized on working with non-human primates. The two animals were healthy at the conclusion of our study and were subsequently used in other studies.

### Task and Recording

**Figure 1:**
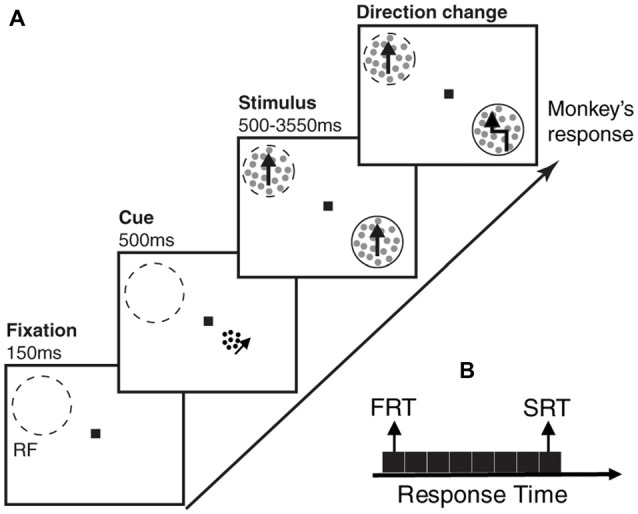
Behavioral paradigm and division of trials based on behavioral response times. **(A)** Each monkey had to foveate on a central point and touch a lever to start the trial. After 150 ms, a cue in the form of a small moving random dot pattern (RDP) appeared close to the fixation point cueing the monkey which of two upcoming moving RDPs to attend to. After 500 ms, the cue disappeared and the two moving RDPs were presented, one of them inside and the other outside the receptive field (RF) of the neuron being recorded. The monkey had to release the lever as soon as the target RDP underwent a brief direction change following a random time between 500–3,550 ms. A trial consists of three time periods; blank period, when the monkey is foveating but no cue has appeared, cue period, when the cue is presented and stimulus period, when the two RDPs are presented but the target stimulus has not yet undergone a change. Dashed-line circles indicate the RF and the solid-line circles show the target position (not shown in the experiment). **(B)** The trials are sorted based on the monkey’s response time and next, partitioned into eight equal-sized subsets. The two subsets with the highest and lowest response times are termed as slow response time (SRT) and fast response time (FRT) classes, respectively in the remainder of the article.

Two male macaque monkeys participated in this study. They were trained to perform a direction change detection task, where they maintained their gaze on a central fixation point and reported a brief direction change in one of two random dot patterns (RDPs; Katzner et al., 2009). The two RDPs moved linearly towards the same direction, either in the preferred or anti-preferred direction of the neuron recorded in cortical visual area MT of the monkeys. To perform this task, animals were seated in a primate chair with their head restrained at a distance of 57 cm from a computer monitor (resolution 40 pixels per degree of visual angle, refresh rate 76 Hz). The eye position was monitored with a high-speed video-based eye tracker at a sampling rate of 230 Hz (ET49, Thomas Recording, Giessen, Germany). Each trial started with depressing a lever and maintaining eye fixation in a window of 1.25° radius, centered on fixation square (0.2° × 0.2°). One-hundred and fifty milliseconds after the lever depression, a cue in the form of a small moving RDP appeared for 500 ms, to indicate the position of the upcoming target stimulus (the stimulus which the animal had to report the direction change of, and which alternated between the two locations across trials of a given session). After the cue disappeared, two moving RDPs (moving towards either the preferred or anti-preferred direction of the recorded neuron) emerged at equal eccentricities in opposite visual hemifields, one of them inside the classical receptive field (RF) of the neuron under study. RDPs moved within a stationary virtual aperture. A single dot subtended 0.1° of visual angle and the dot density was 8 dots/deg^2^. The size of the stimulus RDPs, the speed of the dots, and the direction of motion were matched to the properties of the neuron under study. The cues consisted of small RDPs subtending 0.75° of visual angle, with a dot size of 0.075° and a density of 40 dots/deg^2^. They were always presented at a distance of 2° from fixation, positioned on a virtual line connecting the fixation point to the target location. To make sure the animals were perfectly attending to the cued location, the changes in the stimuli could occur during the next 500–3550 ms succeeding their onset. Trials in which the animal correctly reported the brief change event in the cued location by releasing the lever within a response time window of 100–650 ms, were rewarded by a drop of juice. The trial was terminated instantly after any response (Figure 1A). We divided each trial into three periods; the first 150 ms where nothing was presented (blank period), the next 500 ms during which the cue was presented and the succeeding 500 ms, where the stimuli were shown (before any change occured in either of them).

Using standard surgical techniques (Martinez-Trujillo and Treue, 2004) a Titanium headpost was implanted onto the frontal part of the skull along the midline to allow head stabilization during recording sessions. Additionally, a craniotomy (20 mm diameter) was surgically performed above the parietal lobe and a PEEK recording chamber (Crist Instruments) was implanted. The multi-unit activities (MUA) and LFP signals were recorded form the monkeys’ visual area MT using a five-channel multi-electrode recording system (Mini-Matrix, Thomas Recording) and Plexon data acquisition system (Plexon, Inc., Dallas, TX, USA). The electrode signal was split into LFP and spike components by hardware filters. The LFPs were amplified and digitized at 1 kHz, while spikes were amplified and digitized at 40 kHz. Spikes of the MUA were determined by voltage thresholding. For most of the recording sessions, all electrodes were simultaneously advanced to isolate individual MT neurons with overlapping RFs (linear electrode arrangement, with the impedance of 2 MΩ and 305 μm interelectrode spacing). Cells were characterized as MT neurons based on their directional tuning, RF location, and position in the cortex. The locations and sizes of individual RFs were mapped manually using a moving bar. Direction and speed tuning were determined by presenting a single RDP inside the joint RF, moving in 12 different directions at each of eight different speeds (0.5–64°/s), while the monkeys were maintaining fixation.

### Data Analysis

**Figure 2:**
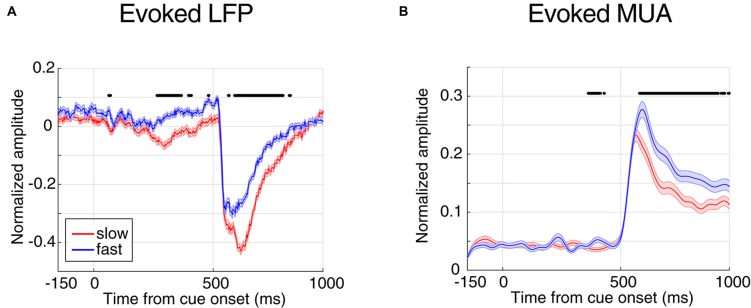
Evoked local field potential/multi-unit activities (LFP/MUA) are predictive of the monkey’s behavioral response time. **(A)** Mean evoked LFP separated by response time. **(B)** Mean evoked MUA separated by response time. Y-axes represent the normalized amplitude of LFP in panel **(A)** and normalized MUA in panel **(B)** and X-axes show the time points aligned to cue onset. FRT trials are shown in blue and SRT trials in red. Horizontal bars indicate those time points with a significant difference in neural activity between the two response time classes (Wilcoxon rank sum test, *P* < 0.00005 for LFP and *P* < 0.001 for MUA).

**Figure 3:**
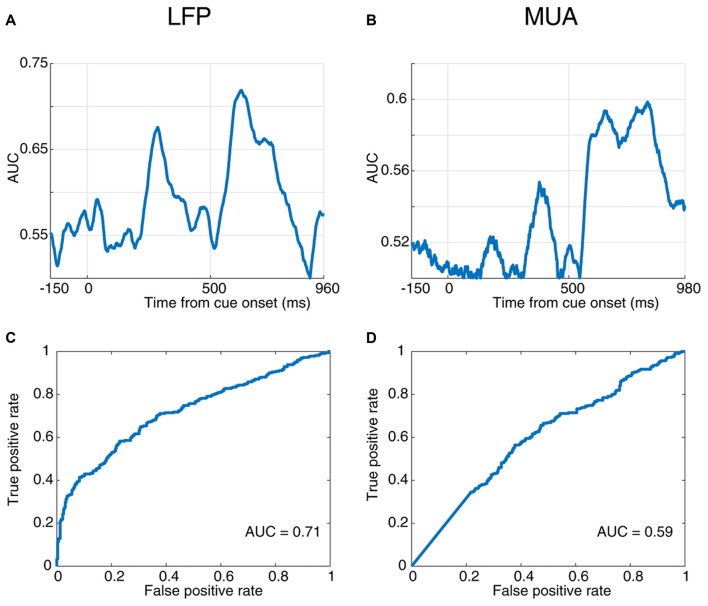
Response time prediction accuracy. **(A,B)** Area under the ROC curve (AUC) values at different times of a trial aligned to cue onset using LFP **(A)** and MUA **(B)**. **(C,D)** Receiver operating characteristic (ROC) curve for the time point with the maximum AUC value using LFP **(C)** and MUA **(D)**.

To analyze the collected data, we only considered the hit trials (trials where the animals reported the target change in the specified response time window—see above) among totally 35 sessions. All trials’ RTs regardless of the target RDP’s location (inside/outside the RF) were taken into account for defining the fast/slow response time. Specifically, we pooled all trials’ RTs together, sorted them in ascending order, divided the resultant vector into eight groups and specified the first and the last group as fast RT (FRT) and slow RT (SRT) trials, respectively. It is noteworthy that our observations were not biased by outlier data. To ensure this, we re-performed the analyses after applying the threshold mean ± 2*STD; we partitioned the RTs into eight classes after removing the trials with RTs less than 240 ms or more than 460 ms. Next, we compared the neural activity (in the form of both LFP and MUA) within the 1st and 8th trial classes. This analysis showed the same results as Figures 2, 3. All analyses were carried out using custom scripts in MATLAB (Mathworks, Natick, MA, USA). The MUA spike trains were convolved with a Gaussian kernel with the σ of 15 ms. In all plots, we first normalized each trial’s neural activity (either LFP or MUA) by the maximum absolute activity across trials of the corresponding site.

### ROC Analysis

In order to examine the neural discrimination across RT conditions, receiver operating characteristic (ROC) analysis was used. This approach clarifies a tradeoff between sensitivity and specificity of classification with the true positive against false positive rate for the different possible cut-off points of test. The algorithm is implemented in three steps: first, fitting a logistic regression model to the neural activity at each time point separately in trials with the response time labels FRT and SRT. Second, based on the resultant logistic regression model, a probability variable (PV) was estimated. Third, by moving the threshold value using the resultant PV, the ROC curve was computed. These steps were performed for all of time points of the signals and the corresponding area under the ROC curve (AUC) for each time point was considered as the accuracy of a binary classification corresponding to that point.

## Results

Two male macaque monkeys were trained to maintain their gaze on a central fixation point and report a brief direction change in the cued (target) one of two moving RDP. The two RDPs moved linearly towards the same direction (either the preferred or anti-preferred direction of the neuron recorded in cortical visual area MT; Figure 1A). Both monkeys successfully reported changes in the target stimulus in more than 80% of the trials (Supplementary Figure S1). LFP and MUA were recorded during the task. Here we investigate if the neural responses evoked by the cue or the RDPs are predictive of the monkey’s RT for reporting a given target change. To this end, we investigate the extent to which LFP and MUA can discriminate the monkey’s RT during different times following cue onset until the behaviorally relevant change occurs.

### Relation Between Neural Activity and Behavioral Response Time

In order to investigate the relationship between the visually evoked neural activity and the monkey’s behavior, we focused on those trials with extreme behavioral response times. We sorted all hit trials based on the animal’s response times and next, partitioned them into eight equal subsets (Figure 1B). Subsequently, the subsets with the shortest and longest response times were considered as FRT and SRT classes, respectively (see “Materials and Methods” section for details).

Next, we compared the evoked neural activity (preceding the direction change) across the two classes of trials with extreme response times. We averaged the time resolved LFP and MUA across trials of each class (Figure 2). The X-axes show the time passed from cue onset and the Y-axes represent the average neural activity across all trials of the experiment, where each trial’s neural activity is normalized by the maximum absolute activity across the trials of the corresponding site. Figure 2A shows the average LFP activity aligned to the cue onset for slow and fast trials (shown in red and blue, respectively). Comparing the average activity across the two response time classes shows that in trials with SRTs, LFPs have a significantly higher evoked absolute activity in the cue and stimulus periods (Wilcoxon rank sum test, *P* < 0.00005, and the two classes become significantly different 265 ms following the cue onset. As expected for the blank period, the LFP response in the two conditions shows no significant difference (Wilcoxon rank sum test, the median *P*-value during the period: *P* = 0.0716). This suggests that the LFP activity evoked by either the cue or the stimuli is predictive of the monkey’s response time. A similar analysis was carried out on the MUA across the two response time classes (Figure 2B). Similar to LFPs, the MUA evoked by the stimuli is significantly different between the two conditions (Wilcoxon rank sum test, *P* < 0.001), diverging 408 ms following the cue (0.16 modulation), and with no significant difference across the two conditions during the blank period (Wilcoxon rank sum test, the median of *P*-value during this period: *P* = 0.6061). On another hand, unlike the LFP, MUA declined any difference in the cue period. This is presumably because the cue is shown outside the RF of the recorded neuron(s), however given that LFP reflects the activity of a larger population of neighboring neurons, the RF of a subset of these neurons might overlap with the cue’s position.

Interestingly, the absolute evoked LFP for FRT trials is smaller than for SRT trials (Figure 2), suggesting that larger evoked LFPs lead to slower responses by the monkey. However, it is the opposite for the MUA; the monkey responds fastest in trials with the highest evoked MUA. This suggests that LFP and MUA in sensory areas play different roles in controlling behavior. We next asked which of evoked LFP or MUA better decodes the monkey’s response time.

### Prediction of Behavioral Response Using the LFP and MUA Neural Responses

Next, to quantify the selectivity of the evoked neural responses to the monkey’s response time, we used a receiver operating characteristic (ROC) analysis. The AUC values are plotted across time for the evoked LFP (Figure 3A) and MUA (Figure 3B), indicating that LFP predicts the RT with up to 72% accuracy and MUA with up to 60% (at 520 ms and 810 ms following the cue onset, respectively). The ROC curve at these two extreme time-points are plotted for LFP (Figure 3C) and MUA (Figure 3D), both showing a significant prediction performance of the response time for evoked LFP (Wilcoxon rank sum test, *P* < 0.00005) and MUA (Wilcoxon rank sum test, *P* < 0.001). Furthermore, LFP outperforms MUA in predicting the response time. Supplementary Figures S2A,B illustrate the average ROC separately for the blank, cue and stimulus intervals.

**Figure 4:**
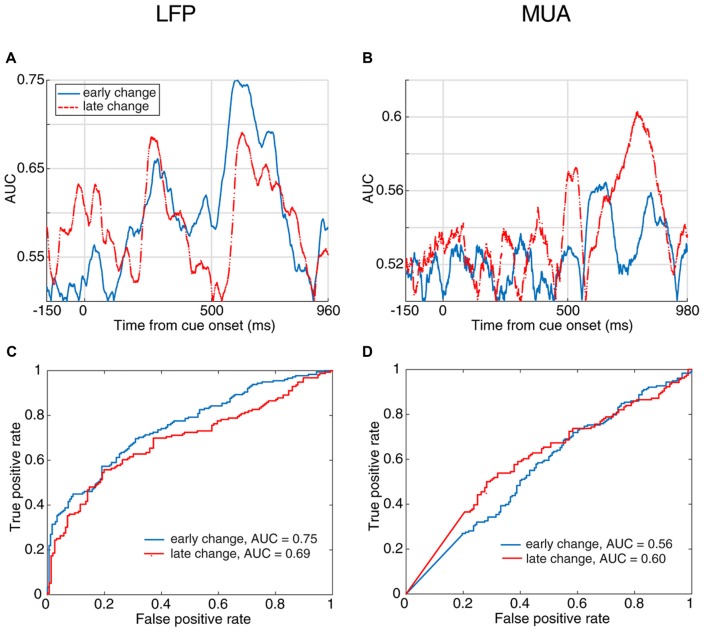
Behavioral response time prediction across different times of change-occurrence. **(A,B)** Prediction of RT from LFP **(A)** and MUA **(B)** in trials with late change time as well as trials with early changes. The enhanced neural activity discrimination between fast and slow behavioral response times even in late change trials, shows that the stimulus-evoked neural activity predicts the monkey’s response to target changes at any arbitrary time. This discrimination was performed for trials with the change between 1,000–2,500 ms as “early change” and “2,500–3,500 ms as “late change.” **(C,D)** Comparison of the prediction performance in early and late change trials based on the ROC curve of the time with maximum AUC for the two change time categories. The red dashed line shows the prediction performance in late change trials and the blue solid line shows the performance in early change trials.

Next, to examine how early the neural activity predicts the response time, we divided the trials into two subsets based on their change times. We considered the trials in which the stimulus change occurred 1,000–2,500 ms following the stimulus onset as “early change,” and trials with their target change occurring 2,500–3,500 ms after stimulus onset as “late change.” Figures 4A,B show the area under the ROC curve across time for discrimination of neural activity between fast and slow RT trials in the early-change and late-change trial categories. Figures 4C,D present the ROC curve for the time with the maximum AUC. These results suggest that the neural activity is predictive of the behavioral RT in both early and late change trials. This reveals that regardless of when the upcoming target change occurs, the stimulus-evoked neural activity (especially LFP) is predictive of the monkey’s speed in detecting the change.

### Position Dependence of the Reaction Time Prediction

**Figure 5:**
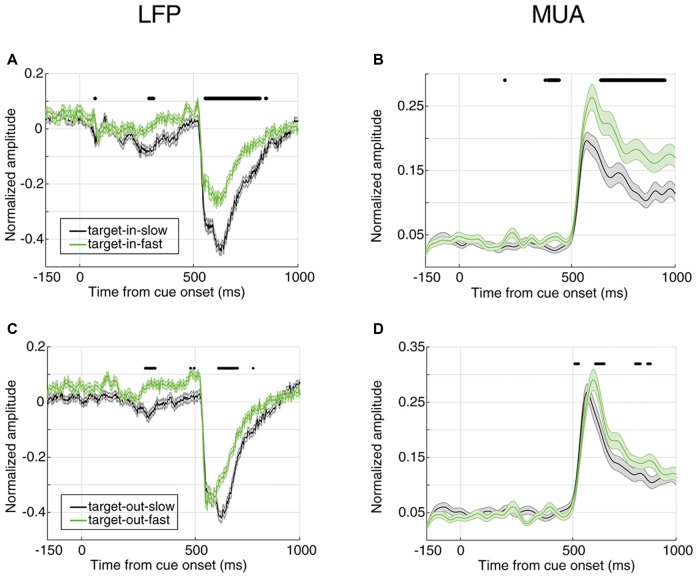
Neural correlate of behavioral response time for different locations of target change. **(A,B)** Average evoked LFP **(A)** and MUA **(B)** for trials where the target change occurred inside the RF. **(C,D)** Average evoked LFP **(C)** and MUA **(D)** for trials where the target change occurred outside the RF. SRT trials are shown in black and FRT trials in green. Horizontal bars indicate the time epochs with a significant difference across response time classes.

So far, we showed that the magnitude of the evoked neural activity predicts the monkey’s response time in reporting an upcoming change. Next, we determined whether this is the case only for changes in the RF or for changes in any position. We first separated the trials where the target change occurred inside or outside the RF (cue pointed to vs. away from the RF, respectively); then, divided them based on the RT (similar to Figure 2) and third, to rule out any effect of the trial frequency, the number of trials in the four conditions (FRT with target change inside RF, SRT with target change inside RF, FRT with target change outside RF, SRT with target change outside RF) were equalized. Figure 5 shows the average evoked LFP and MUA for each of the two conditions of target position (inside/outside RF) in the two response time classes (FRT vs. SRT). The top panel shows the average LFP and MUA for trials where the target change occurred inside the RF and the bottom panel shows the evoked neural activity for trials where the target change appeared outside the RF. In both types of trials where the target change occurred inside or outside the RF, the average LFP/MUA responses evoked by the stimuli differed significantly between the response time conditions (Wilcoxon rank sum test, *P* < 0.00005 for LFP, *P* < 0.001 for MUA). However this difference is clearly larger (for both LFP and MUA) when the target change occurred inside RF (Figures 5A,B) compared to outside RF (Figures 5C,D).

**Figure 6:**
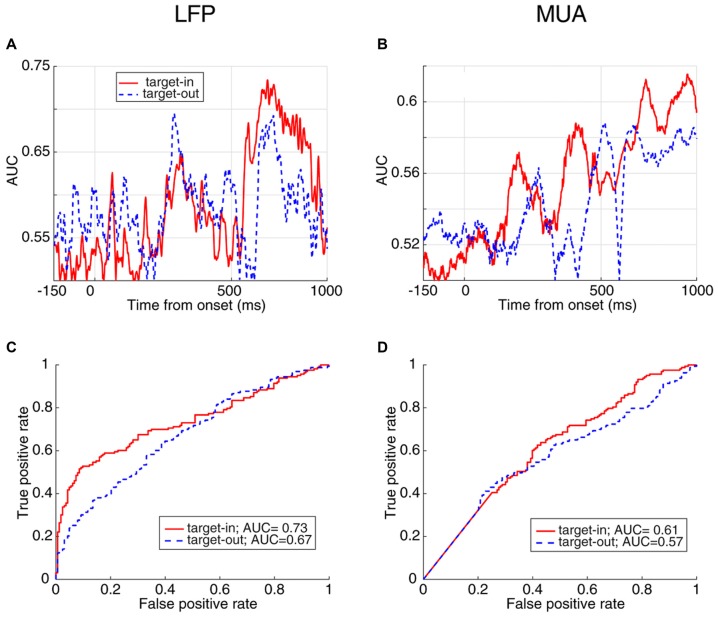
AUC and ROC curves for FRT/SRT classes separated by target position. **(A,B)** The AUC values at different times for the two classes, FRT and SRT in target-in (red) and target-out (blue) trials using LFP **(A)** and MUA **(B)**. **(C,D)** ROC curve of the time point with the maximum AUC plotted for target-in (red) and target-out (blue) conditions for LFP **(C)** and MUA **(D)**.

In order to compare how distinct the response times are between the two target change locations, we calculated the instantaneous AUC and the ROC curves for trials where the target change occurred either inside or outside RF (Figure 6). Figure 6A shows the AUC across time for target-in (red) and target-out (blue) conditions using LFP signals, where each point plots the AUC for a 20 ms time window. For the stimulus period, the target-in condition shows a higher response time discrimination compared to the target-out condition. Similarly, MUA shows higher AUC values in the target-in compared to the target-out condition during the stimulus period (Figure 6B). Along the same lines, the ROC curves at the time point with the maximum AUC (Figure 6C for LFP and Figure 6D for MUA) are both indicative of a higher discrimination accuracy in the target-in rather than the target-out condition (Wilcoxon rank sum test, *P* < 0.05; see Supplementary Figures S2C,D for the average ROC across the time-epochs of a trial for target-in and target-out conditions and Supplementary Figure S3 for the neural discrimination separated by the target change location). These results suggest that: (1) the stimulus period provides the strongest prediction about the response time, compared to the preceding epochs; (2) the evoked neural activity (either LFP or MUA) is mostly reflective of the response time to changes inside the RF, rather than other positions in the visual field, indicating that this evoked neural activity is a local, rather than a global predictor of behavioral performance.

We were concerned that the difference between the neural activities across response time classes is due to a difference in the distribution of sites each class comes from. This issue is particularly important since different sites are recorded from in different sessions (recording days), making the corresponding trials vary in terms of the animal’s average response time. To rule this out, we focused on those sites which exclusively provided either FRT or SRT trials. By comparing the trials coming from each of FRT-providing and SRT-providing sites (Supplementary Figure S4A; excluding those originally considered as FRT and SRT trials; to ignore the response time associated effect), we observed no significant difference between the neural activity of the two trial sets (Supplementary Figure S4B). This suggests that the two sets of sites associated to either SRT or FRT trials do not differ in terms of their general physiological properties. Therefore, the neural activity difference between FRT and SRT trials is not induced by differences between the distribution of sites (via their physiologically different properties) corresponding to each trial type.

We further examined if the neural discrimination of RT is not limited only to the 1st/8th clusters, with the other six clusters of trials (the remaining 75% of the data) not showing any neural activity-RT association. This concern becomes particularly important considering that the data in these clusters might be biased by outliers. We therefore, considered also the clusters in between and plotted their neural activity (both LFP and MUA) separately (Supplementary Figure S5). The traces show a rather monotonous change of the amplitude of neural activities between the clusters, indicative of a functional association between the magnitude of neural activity and RT. Consequently, stimulus-evoked neural activity is selectively predictive of the monkey’s behavioral speed in detecting the visual change presented inside the neuron’s RF.

## Discussion

We reported here that visually evoked LFP and MUA in macaque MT are predictive of how fast primates report a visual change. We found that first, both visually evoked LFP and MUA can predict the response time of monkeys in detecting brief visual changes on a trial-by-trial basis. Second, the prediction performance calculated using LFP is significantly higher than MUA. Third, visually evoked responses (for both LFP and MUA) have enhanced information on the response time to the change inside, rather than outside the RF of the recorded neurons.

Our finding of the association between evoked neural activity and behavioral response time is consistent with previous studies showing a direct link between population neural responses and behavioral response for neurons in visual cortical area MT (Britten et al., 1996; Liu and Newsome, 2005; Cohen and Newsome, 2009). In line with our results, a previous study based on event-related potentials (ERPs) has shown that the amplitude of the first negative component of the ERP (N1) has a negative correlation with RTs (Toledo et al., 2015). Although some reports suggest that the response times to visual or auditory stimuli are linked to the neural activity in brain regions associated to response initiation (i.e., motor-related potential; Yordanova et al., 2004) and motor preparation (Antonova et al., 2016), our results suggest that variability of the response times is highly predictable by the neural activity in sensory areas. The correlation of RT and neural activity could be either due to a simultaneous influence of spatial attentional influences on both RT (Posner, 1980) and neural activity (Katzner et al., 2009), or due to a causal influence of evoked neural activity on the behavioral performance.

Furthermore, the neural discrimination between fast and slow trials is present when the target stimulus is presented inside, rather than outside the RF (see Figure 5). This is further consistent with previous studies addressing ERPs which show the expectancy of a specific cued target could be handled at three levels of sensory, attention, and motor preparation (Wright et al., 1995). Importantly, the grown difference between the neural activities across the two RT conditions Target-in-FRT vs. Target-in-SRT (see Figures 5A,B) during the stimulus period (time interval 500–1,000 ms) seem to be a consequence of a successful combination of the three cognitive processes outlined above. Consistently, the discrimination between the neural activities of the two RT conditions Target-out-FRT vs. Target-out-SRT (see Figures 5C,D) was significantly smaller compared to those trials were the stimulus change occurred inside RF even during the stimulus representation interval where the sensory response is maximized. This indicates that neural activities could predict how well a visual stimulus is processed in a spatially localized manner. This is particularly important in brain computer interface applications where predicting the human response to a spatially specific visual stimulus is desired.

We found that a larger absolute evoked LFP precedes a slower behavioral response while a smaller evoked LFP is followed by a faster response. For the MUA we found that a larger evoked activity precedes a faster behavioral response, while a smaller evoked MUA is associated with a slower response. This suggests that the evoked MUA’s amplitude can serve as a neural readout of the sensory cortex’s capacity for processing the upcoming stimulus and therefore representing its upcoming change. However, for the LFP our results show the opposite pattern; a higher amplitude of the evoked LFP predicts a less effective processing of the upcoming change. This suggests that the evoked LFP’s magnitude is linked to the sensory neural network’s unresponsiveness in terms of processing changes. Since LFP reflects mostly synaptic potentials of neurons, this means that the stronger the synaptic potentials induced in the local neural network, the less efficient the upcoming stimulus change is processed, reflected also by a smaller MUA amplitude. This is consistent with a previous analytical study, suggesting an association between the noise level and signal amplitude of neural inputs (Shomali et al., 2018). Our observation suggests a different readout of the sensory areas’ sensorimotor capacity by LFP and MUA, inline with our previous report documenting a time difference between visually evoked LFP and MUA’s peaks (Esghaei et al., 2017).

To conclude, our data suggest that: (1) LFP and MUA in are a MT are predictive of the monkey’s speed in reporting a visual change up to 2,000 ms preceding it; (2) the stimulus-evoked neural activity’s discrimination of response speed is a local phenomenon specific to when the target change occurs inside the RF; (3) LFP amplitude has a negative correlation, while MUA is positively correlated with the behavioral response speed. Our findings may aid both brain computer interaction applications in predicting a human’s intention using online recordings of neural activities, and enhance the performance of human-robot interaction systems, by allowing robots to know the human decision way before it is made.

## Data Availability

The datasets generated during the current study are available from the corresponding author on reasonable request.

## Author Contributions

MPD, MBK, ST, MRD and ME designed the study. MPD and MBK performed data analyses. MPD, MBK, ST, MRD and ME interpreted the data and revised and approved the articles’ final version. MPD, MBK and ME wrote the initial draft of the article.

## Conflict of Interest Statement

The authors declare that the research was conducted in the absence of any commercial or financial relationships that could be construed as a potential conflict of interest.
