# Supplementary material for: Neural Activity Predicts Reaction in Primates Long Before a Behavioral Response
Source: Front Behav Neurosci. 2018 Sep 11;12:207. doi: 10.3389/fnbeh.2018.00207 (PMC6146178; doi:10.3389/fnbeh.2018.00207)
Supplement: Supplementary file 1 [file Data_Sheet_1.pdf]

Supplementary Material

Neural Activity Predicts Reaction in Primates Long Before a Behavioral Response

Mohsen Parto Dezfouli[¥], Mohammad Bagher Khamechian[¥], Stefan Treue[♯]*, Moein Esghaei[♯], Mohammad Reza Daliri[♯]*

[¥] co-first author

[♯] co-senior author

*Correspondence should be addressed to:

Stefan Treue (treue@gwdg.de)

Mohammad Reza Daliri (daliri@iust.ac.ir)

Supplementary Figures

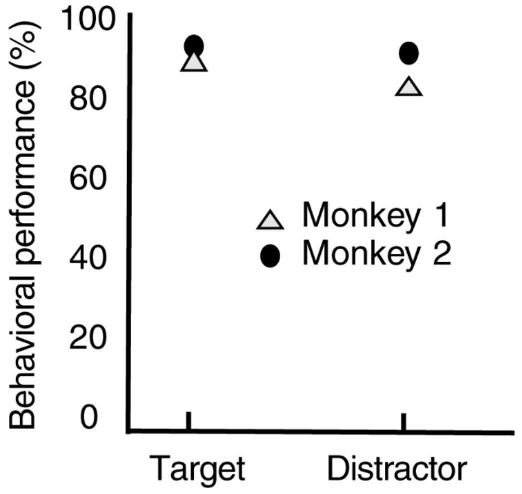

Supplementary Figure 1. Average performance of the two monkeys in reporting the target change (Target) or ignoring the distracter change (Distractor).

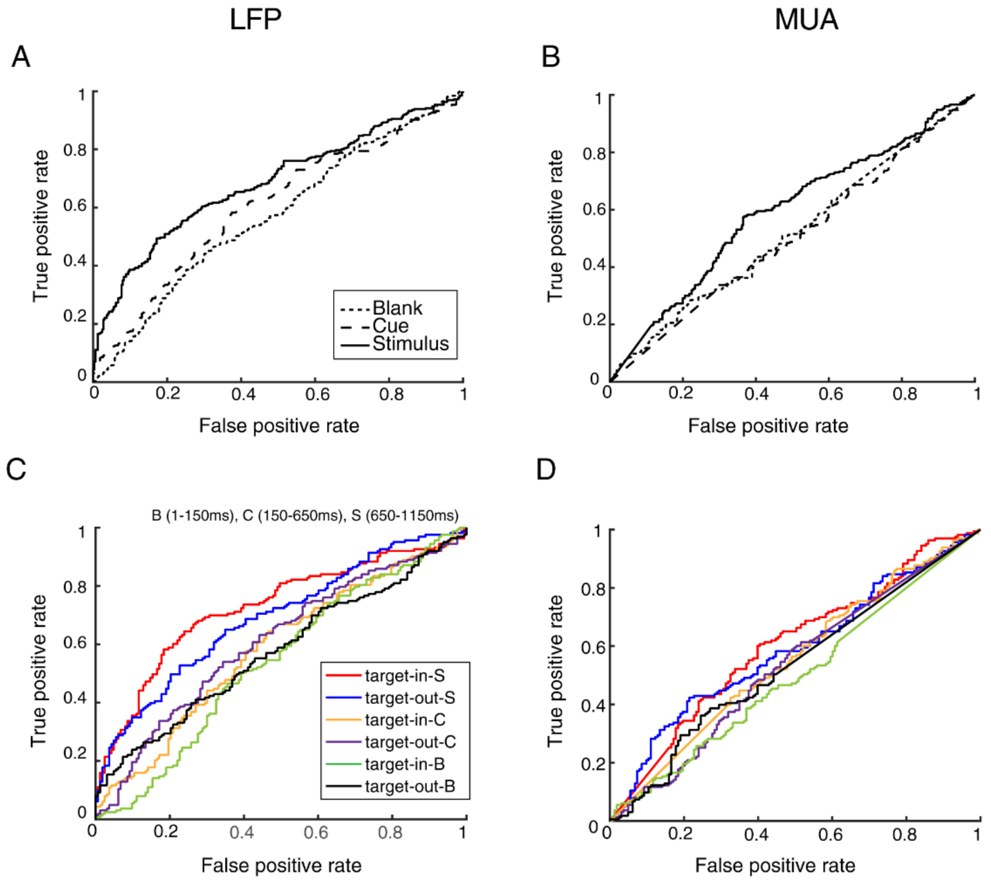

Supplementary Figure 2. ROC analysis across the three task periods. (A, B) ROC curves, separately for three task periods; Blank (1-150ms), Cue (150-650ms) and Stimulus (650-1150ms) for LFP (A) and MUA (B). (C, D) ROC curves of the three periods based on LFP (C) and MUA (D) for target-in and target-out conditions, separately. The characters B (blank period), C (Cue period) and S (stimulus period) show the task periods used for the ROC calculation.

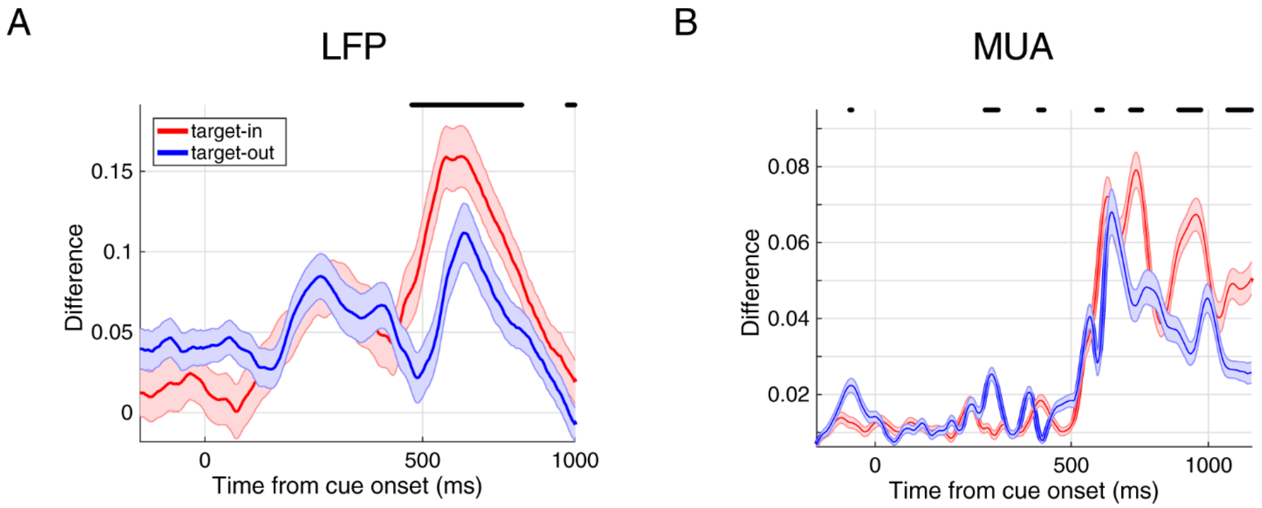

Supplementary Figure 3. Neural signature of reaction time depends on the location of target change. (A) The LFP's difference between fast and slow response times, time-resolved in target-in (red) and target-out (blue) conditions. The response differences were smoothed with 150ms averaging windows. (B) The MUA difference between fast and slow response times, across time in target-in (red) and target-out (blue) conditions. For each panel, we randomly selected 20% of trials from each class of fast and slow trials in each target change location. We next subtracted the average neural activity within each of the target-in and target-out conditions, across the reaction time classes. Error bars represent the standard error of mean across 50 repetitions.

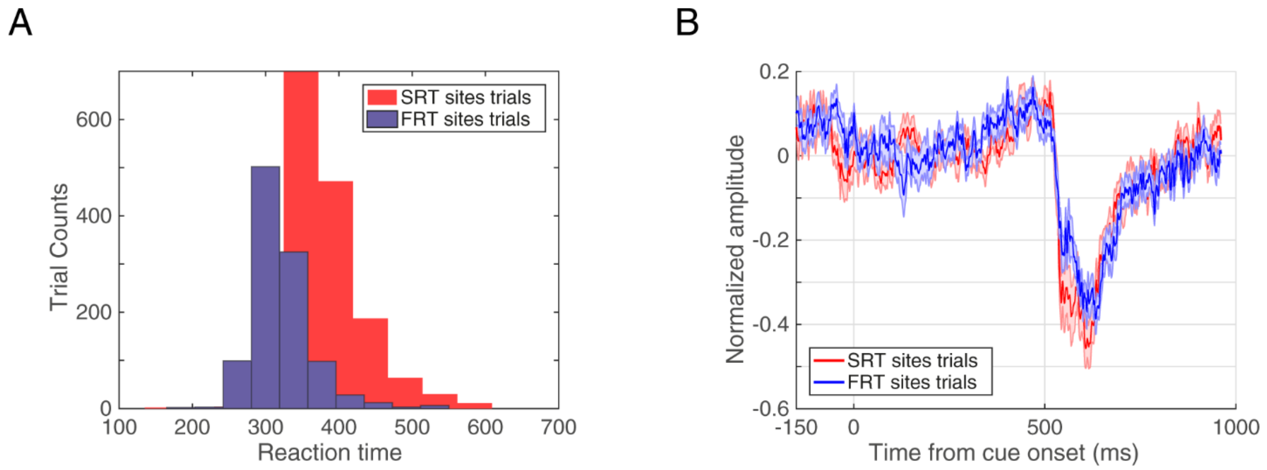

Supplementary Figure 4. Control for recording sites. (A) Histogram of RTs, separated by recording sites for which only fast trials or slow trials (FRT sites trials and SRT sites trials, respectively) were considered. In order to verify if our observed difference between fast and slow trial groups is not due to their coming from different recording sites, we selected two different subsets of trials corresponding to the same set of sites, but without a response time difference. Comparing the two trial subsets' average LFPs shows no significant difference between the two (B). This suggests that the neural activity difference between FRT and SRT trials is not induced by systematic physiological differences across the corresponding sites.

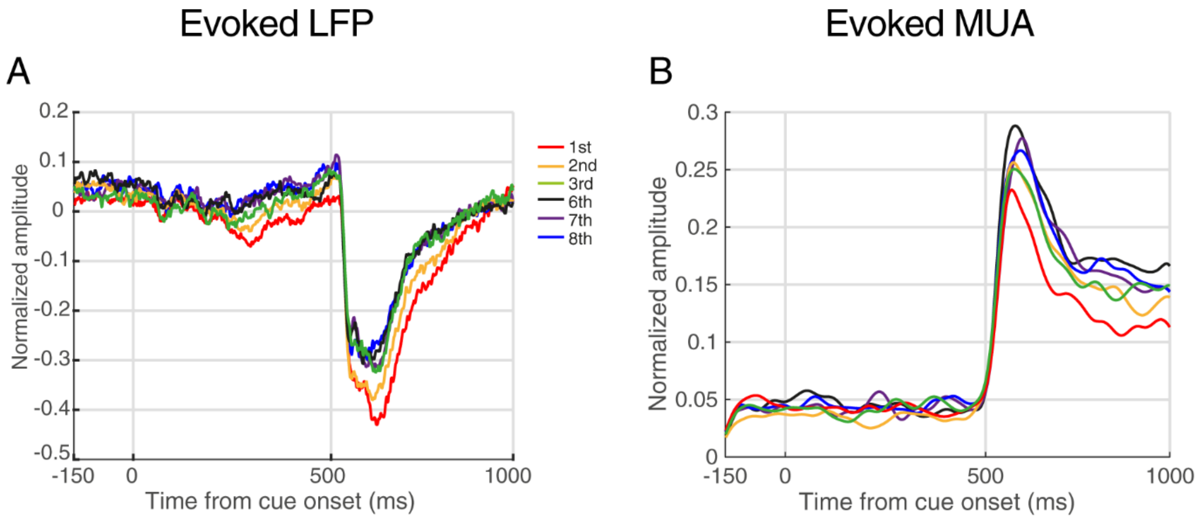

Supplementary Figure 5. LFP and MUA in middle trial classes. (A, B) To find the dynamics of LFP and MUA responses in trial classes (sorted relative to their RTs) other than the extremes (1st and 8th), we computed the average of normlized LFP and MUA responses for the 2nd, 3rd, 6th and 7th classes of RTs. The average evoked LFP (A) and MUA (B) for these four quantiles is plotted together with that of the 1st and 8th quantiles. The systematic response time dependence of the evoked activities even within the middle trial classes indicates that the observed neural discrimination of RT is not due to an outlier effect. X and Y-axes are the same as figure 2.
